# A New Trick for the Venotomy in Neonates

**DOI:** 10.21699/jns.v6i2.560

**Published:** 2017-04-15

**Authors:** Stefano Benvenuti, Filippo Parolini, Daniele Alberti

**Affiliations:** 1 Department of Paediatric Surgery, Spedali Civili Children’s Hospital, Brescia, Italy; 2 Department of Clinical and Experimental Sciences, University of Brescia, Bresica, Italy

**Dear Sir**

The management of Low Birth Weight (LBW) and Very Low Birth Weight (VLBW) neonates needing intensive cures usually requires an intravenous line for total parenteral nutrition (TPN), blood and platelet transfusions, antibiotics administration and blood samplings. In this respect, the use of central venous catheters (CVC) has become mandatory in neonatal intensive care units (NICUs) [1-2]. Peripherally Inserted Central Catheters (PICCs) are the most common used device by the neonatologists facing LBW and VLBW neonates [2-3]. When these patients develop surgical complications or need of high volume liquid infusion or exchange blood transfusions, or multi drugs administration, percutaneous ultrasound-guided CVC placement through the jugular, subclavian or right brachiocephalic vein represents the treatment of choice [1-3]. However, the experience with percutaneous CVC placement in VLBW newborns is limited. When large CVC are needed, their surgical placement still remains a common procedure in NICUs [4]. For its superficial position and satisfying caliber, saphenous vein is one of the most common used vein [4]. Because of dissection of the surrounding tissue, once identified and exposed, saphenous vein usually collapses that makes its cut-down very challenging, particularly in these very small babies. With scissors kept oblique or perpendicular to vessel’s course, an opening must be create wide enough to allow a smooth introduction of the catheter or at least the insertion of one of the scissor jaws to open the lumen with a second longitudinal cut to the intima. Damaging these very tiny and fragile veins, even up to the extent of cutting the posterior wall, encumbers this step of the procedure. As these complications are not uncommon, we conceived a new trick to safely cut- down these veins, successfully performed in more than 300 procedures in the last 20 years.

A 22-24G insulin needle is bent at right angle by a clamp for about 1 cm (Fig.1A). Once the saphenous vein is exposed, encircled and loaded over threads, two stay suture including the subcutaneous tissue, the superior edge of skin incision and the skin over the inguinal crease are placed in order to expose the operative field and to leave the assistant with both hands free (Fig.1B). With the needle cut surface facing up, the vein is punctured in a parallel way to its course. Paying attention to always stay parallel to the vessel’s course, the needle is gently advanced into the vein for 5-7 mm and then retracted (Fig.1C). Just before the needle exits the vein, it is gently pushed upwards to open the wall of the vein from the inside outward at the needle entrance site, in order to enlarge the hole. One of the scissors’ jaw is then easily introduced through this hole and the vein is longitudinally cut, staying parallel its course (Fig.1D). Vein cutting edges are then clearly visible and the insertion of the catheter becomes very safe and easy. Eventually, the vein is tied above and below the catheter entrance with an absorbable monofilament.

**Figure:**
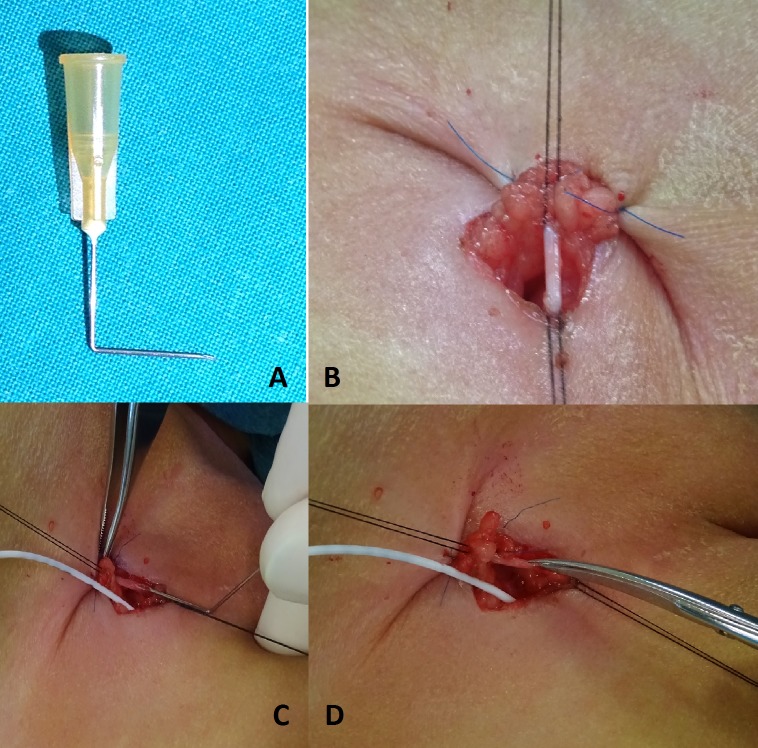
Figure 1: Steps of venous cut down.

## Footnotes

**Source of Support:** Nil

**Conflict of Interest:** None
